# The Horse on the Prairie

**Published:** 1882-02

**Authors:** R. J. Burdette


### THE HORSE ON THE PRAIRIE.

WHILE we trotted along, a young farmer came galloping over the prairie to us. Now, if you have never seen the horse on the prairies, you have never seen him at all. He belongs there. Until you see him in his home, you can never realize how tame a picture he makes curveting in the streets of a city, or prancing through the thronged drives of a park. But out here—the day is full of sunshine, the air is bracing and pure, and on these plains it is exhilarating as champagne. As far out on the pale brown prairie as you can distinguish objects you can see the moving speck on the horizon, and watch it coming into clearer view as you see a ship sail in sight at sea. The figure of the man and horse seem one; the motion of the easy gallop is regular as music, rising and falling in perfect cadence. As they come nearer, the figure of the horse, perfect in outline and graceful in every movement, the long tossing mane, the easy seat of the rider, riding with straight knees and long stirrups, and by-and-bye the muffled flutter, rather than clatter of the hoofs on the turf, and back of and around all this the background of a far-reaching prairie dimpling in all shades of brown, and the setting of a sky blue as turquois, with the wide, wild sense of perfect freedom, a universe in sight, makes a picture that you never want to forget, and could not forget it if you would. We all wanted to shout as the rider galloped up, and, with a cheery "Hello" to our driver, went swinging on. I have seen beautiful saddle horses in Fairmount park, and I have watched riders in Central park pounding their saddles with the trip-hammer ease of the English riding-school; I have seen the "flyers" and their wonderful jockeys, throwing the miles away like so many seconds in Jerome; I have seen armies of cavalry sweep across the battle-field, while the ground fairly rocked and trembled under their charging feet; I have watched, thrilled with excitement, a six-gun battery go wheeling and thundering into position in the very face of a charging column at a time when minutes seemed hours, but I think I never saw the horse when he seemed so much a part of the landscape; when all the freedom and beauty of earth and air and sky seemed to be made to harmonize with him, his strength and beauty and grace, until I watched him sweeping over the great sky-encircled prairies of the West.—*R. J. Burdette.*

